# Agent-based social simulations for health crises response: utilising the everyday digital health perspective

**DOI:** 10.3389/fpubh.2023.1337151

**Published:** 2024-01-17

**Authors:** Jason Tucker, Fabian Lorig

**Affiliations:** ^1^Department of Global Political Studies, Faculty of Culture and Society, Malmö University, Malmö, Sweden; ^2^Department of Computer Science and Media Technology, Malmö University, Malmö, Sweden; ^3^Internet of Things and People Research Center, Malmö University, Malmö, Sweden

**Keywords:** agent-based social simulations, health policy, crisis, COVID-19, everyday digital health, artificial intelligence

## Abstract

There is increasing recognition of the role that artificial intelligence (AI) systems can play in managing health crises. One such approach, which allows for analysing the potential consequences of different policy interventions is agent-based social simulations (ABSS). Here, the actions and interactions of autonomous agents are modelled to generate virtual societies that can serve as a “testbed” for investigating and comparing different interventions and scenarios. This piece focuses on two key challenges of ABSS in collaborative policy interventions during the COVID-19 pandemic. These were defining valuable scenarios to simulate and the availability of appropriate data. This paper posits that drawing on the research on the “everyday” digital health perspective in designing ABSS before or during health crises, can overcome aspects of these challenges. The focus on digital health interventions reflects a rapid shift in the adoption of such technologies during and after the COVID-19 pandemic, and the new challenges this poses for policy makers. It is argued that by accounting for the everyday digital health in modelling, ABSS would be a more powerful tool in future health crisis management.

## 1 Introduction

During the COVID-19 pandemic, there was a momentous increase in interest in how artificial intelligence (AI) systems could be used to manage the crisis (1, 2). Policy makers faced a huge challenge of having to try to analyse the potential consequences of different policy interventions, doing do with very limited time, recourses and tools to assess the impact or potential outcomes. Compounding these challenges, was the issue that, while pandemics are not new, the COVID-19 pandemic posed unique challenges for policy makers (3). As such, there was a limit to the usefulness of drawing on past lessons on pandemic management. At the same time the pandemic saw a surge in the adoption of digital health technology to overcome the pressure on health services and lack of mobility many people faced, with this adoption having varying levels of success (4–6). The use of digital health such as websites, social media, telemedicine, patient self-care devices, health apps, wearable tracking devices, persuasive computing and diagnostics, to name but a few, fundamentally altered health and healthcare for many people, doing so in ways that we still do not understand.^1^

Within this context, agent-based social simulations (ABSS) proved to be very well suited to informing policy decision making (7, 8). ABSS aim to model and simulate the actions and interactions of intelligent agents^2^, creating virtual populations or social systems composed of autonomous (artificial) individuals (11). These virtual societies can serve as a “testbed” for investigating and comparing different policy interventions and scenarios prior to their implementation. While ABSS cannot predict the future, it is a powerful tool to help inform policy makers of potential outcomes or consequences of interventions. ABSS enables decision makers to “play” with policy, and variations of policies, and investigate their impact under different circumstances and scenarios (12). Doing so in a virtual population allows for the conducting of experiments in a time and cost-efficient manner, removes the risk of harming real-world individuals and can facilitate greater levels of preparedness and response to health crises.

However, while ABSS is not a new technology its application in the policy making space is still in its early days, with its implementation during COVID-19 not being without a certain amount of friction. This piece focuses on two prominent points of friction that were identified, these being defining valuable scenarios to simulate and a lack of appropriate data (13). This paper explores how we can reduce these frictions if an “everyday” digital health perspective is adopted in the development of ABSS for health crisis management. It is claimed that by recognising the adoption and normalisation of digital health technologies (and crucially how and why this is experienced differently by various groups) can strengthen ABSS, positioning it as a powerful tool in manging health crises.

## 2 ABSS and COVID-19

*“Simulation is the imitation of the operation of a real-world process or system over time”* (14).

Computer simulations provide a virtual environment for conducting experiments with a target system and can be used to analyse the behaviour or dynamics of that system under different circumstances (15). Similar to real-world experiments, this allows us to conduct *what-if* analyses and to explore different scenarios to investigate how changes to the system’s configuration or exogenous factors might affect the system’s overall behaviour. A major advantage of using a virtual environment is that the experiments do not risk jeopardising the system under investigation. Simulation experiments allow for more time and cost-efficient analyses and enable the generation and investigation of situations or conditions that might rarely occur, e.g., crisis or disaster situations. This type of experiment is also referred to as *in silico experiment* and is increasingly used in, for instance, biology, social sciences, and engineering (16, 17).

There exists a variety of applications where computer simulations are used in healthcare (18, 19). Particularly during the COVID-19 pandemic, a great number of simulation models were been developed to investigate infection dynamics and the effects of non-pharmaceutical interventions (12, 13, 20). Traditionally, epidemiological models make use of differential equations and transition probabilities to describe the dynamics of an entire population of individuals, neglecting individual infection statuses and infection chains. For simulating the consequences of specific non-pharmaceutical interventions or other policy measures, however, individual behaviour needs to be taken into account as the success of these intervention greatly depends on how and to what extent each individual adapts to and comply with instructions and restrictions.

A simulation paradigm that is particularly well suited to simulate individual behaviour is ABSS. ABSS make use of an artificial population of autonomous individuals, so called *Agents*, each of which is characterised by a set of attributes, e.g., age, gender, and health status. Based on these personal attributes, its environment, and its individual needs and goals, each agent individually plans its actions by imitating human-like behaviour using AI (10). The goal of ABSS is to imitate the relevant aspects of the real-world population, i.e., composition and behaviour, as closely as possible to allow for drawing sound conclusions regarding the target system.

Traditional simulation approaches in healthcare and epidemiology aim to directly model the dynamics of the phenomenon of interest. In ABSS, however, the system’s behaviour and the corresponding macro-scale phenomena emerge from micro-scale agent behaviour. This allows not only for analysing *what* potential consequences a given scenario or intervention might result in but also provides a better understanding *why* certain effects can be observed. Gilbert and Troitzsch (21, p. 1) argue that individual-based simulations imply a “new way of thinking about social and economic processes,” due to the emergence of complex behaviour from simple actions and interactions. ABSS can be applied to review theories, to verify assumptions, and to generate data and can therefore, according to the authors, be considered a new method of theory development.

In the early phase of the COVID-19 pandemic, policy makers were supposed to make critical decisions facing rapid developments and incomplete data. Identifying appropriate policy interventions under such a high degree of uncertainty is challenging and sound decisions require considering different sources of information and evidence. To address these challenges and to assess uncertainty, ABSS of the COVID-19 pandemic were developed and applied (22). By modelling an artificial population and presumed transmission processes, the possible effects of different interventions (e.g., lockdowns, social distancing, or facemasks) under different possible scenarios (e.g., in different countries or at different stages of the pandemic) could be simulated to inform policy makers of their potential outcomes. The use of ABSS to inform policy making has gained popularity, and other recent application areas include disaster management (23), healthcare (24), agriculture (25) and transportation (26).

**Figure 1:**
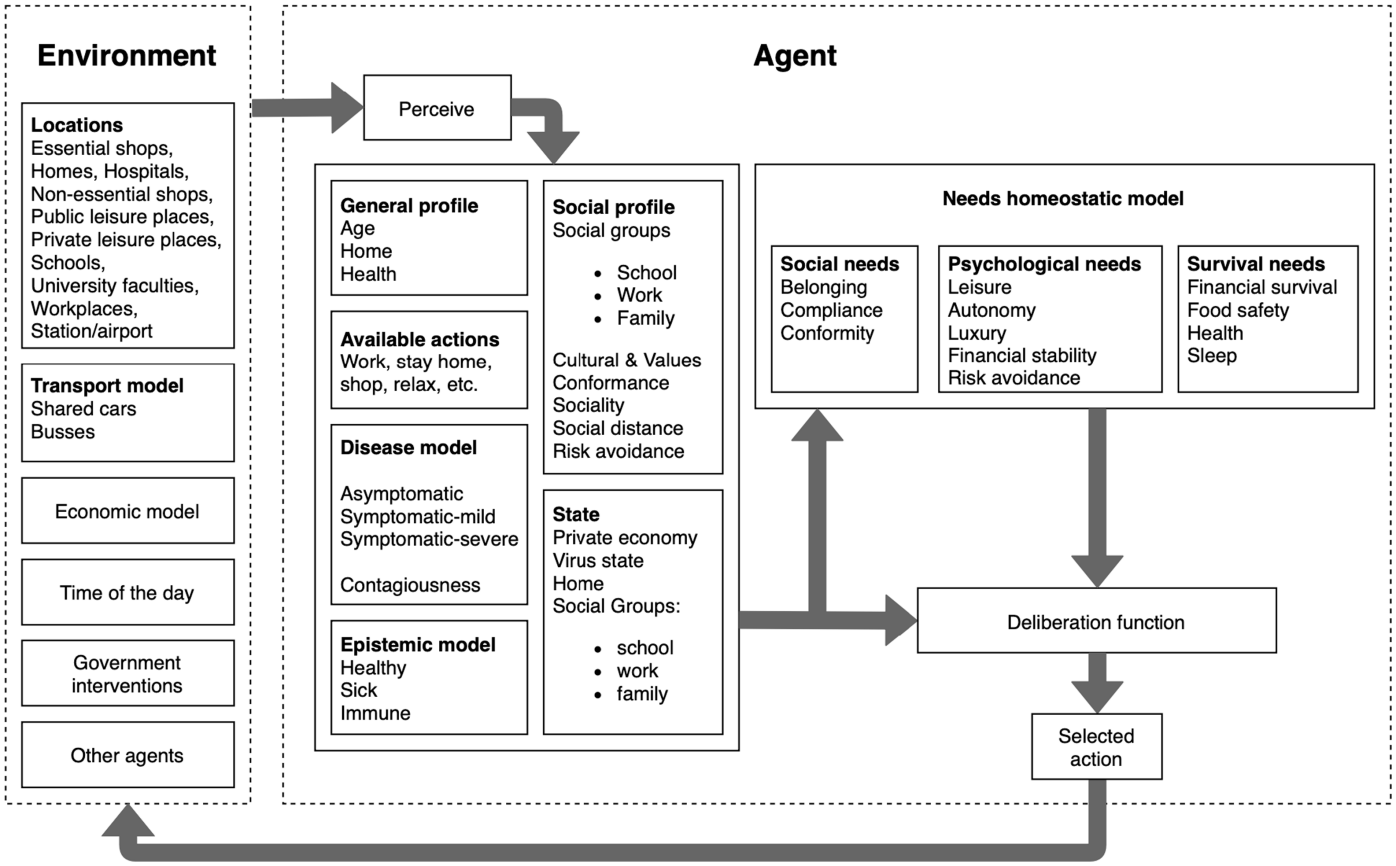
Architecture of the ASSOCC simulation model (29).

The ASSOCC model (27) is one example of an ABSS that was developed in the early phase of the COVID-19 pandemic for analysing the effects of different non-pharmaceutical interventions and to inform policy making. The agents’ behaviour is based on individual needs, which are inspired by Maslow’s hierarchy of needs, and the satisfaction of these needs through specific actions promotes different values, according to Schwarz theory of basic human values (28). In the ASSOCC model, the satisfaction of needs is implemented using a water tank approach, where each need is represented by a water tank depleting over time and the agent will try to keep their tanks filled up by pursuing appropriate actions. The model further consists of a representation of different locations (e.g., homes, hospitals, schools, and workplaces), an economic model, a transport model, and time progress is modelled by dividing the day into four phases (i.e., morning, afternoon, evening, and night). The architecture of the ASSOCC simulation model is shown in Figure 1.

**Figure 2:**
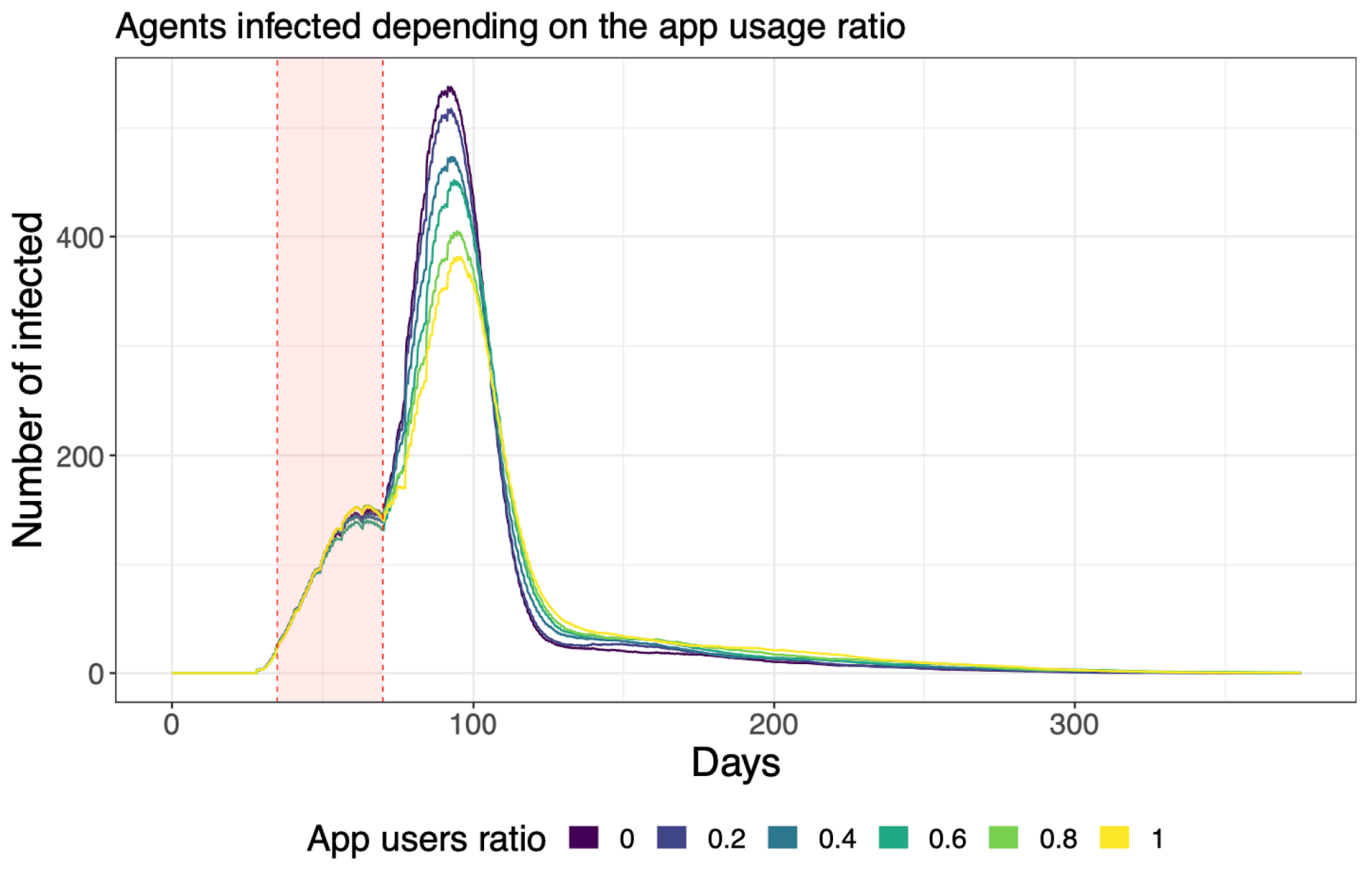
Number of infected agents for different levels of app usage in the simulation (30).

One of the interventions that can be simulated using the ASSOCC model are tracking and tracing apps (30). The implementation of these apps was been discussed by different countries at an early stage of the pandemic (31). By tracking contacts with other persons using mobile phones, infected individuals can, once they are diagnosed with COVID-19, inform those they have met during the last days such that they can isolate themselves. The simulation results indicated that higher numbers of app users can indeed decrease the peak of infections, however, the effects are marginal even in moderate app usage scenarios, which is due to contacts with other persons shifting from public to private places (see Figure 2).

## 3 The “everyday” digital health perspective

This everyday perspective on technology^3^ highlights that technology does not exist independently of humans. It warns against seeing humans as without agency and society as merely a site upon which technology is dropped from above (33). The everyday pushes back against the tendency to reduce people to data points (itself very problematic), or ignoring individuals’ agency, approaches which are prone to inaccuracy (*ibid*). In adopting an everyday perspective on digital health, we are reminded to focus on “weaker” actors who may not normally be considered as being important players in transforming global contexts through their local actions (34). This everyday approach calls for the recognition that a broad range of actors are considered as interacting with digital health, not just the technology developers, but the patients (consumers,) carers, practitioners etc. (*ibid*). The everyday perspective centralises and embraces the reality that digital health exists within complex, fluid, and co-constitutive everyday spaces where people and technology interact.

Foregrounding the everyday digital health perspective in policy development is crucial if we are to be able to best develop policy for the implementation of digital health applications and improve health outcomes. However, in crises contexts, with severe limitations on time and resources, doing so is a challenge. The richness of the everyday perspective of digital health, with its focus on the highly individual, fluid, deeply complex and context dependent co-constitutive relations means there is a vast but disjointed body of research. The need to connect this data is essential: fragmented data was one of the main limitations with policy making during COVID-19 (35). ABSS with its focus on individual agent autonomy is well suited to using the fragmented micro (everyday) level to inform the macro level. It is argued here that doing so would not only elevate the existing research on the everyday, but would address two significant challenges of using ABSS in future health crises.

## 4 ABSS and the everyday digital health perspective

Even though a great number of ABSS models were developed during the COVID-19 pandemic, policy actors still seemed hesitant to use them to inform their decision-making processes (36). There was also a mismatch between the developed models and the information decision makers actually needed for facilitating and supporting their work (13). Developing appropriate and useful models is a joint effort and requires a close collaboration between modellers, policy actors, and other stakeholders. Based on a survey of collaborations between ABSS modellers and policy makers, Belfrage et al. (36) identify five challenges that commonly occur including disagreement regarding what scenarios to model, unrealistic expectations in terms of the contribution of simulations, lack of stakeholder engagement, lack of technical understanding, and general scepticism regarding the generated results. We focus on two key challenges faced when implementing ABSS during the COVID-19 pandemic, defining valuable scenarios to simulate and the lack of appropriate data, and how the everyday digital health perspective can, in part, address these.

### 4.1 Defining valuable scenarios

In ABSS, a scenario is a specific configuration of the model that can be used to investigate a particular situation or phenomenon. It consists of the input data that is used to initialise the model, the parameter values to adapt the model to the specific circumstances, the synthetic population, and different assumptions regarding the underlying mechanisms. For instance, when analysing a COVID-19 intervention, such as lockdowns, a scenario must be developed that specifies the length and extent of the lockdown but also the composition of the population and the time horizon of the simulation. In many ABSS of the COVID-19 crisis, lockdowns were modelled as the entire population not leaving their homes, resulting in a rapid decline in infections. In most countries, however, lockdowns were associated with certain exceptions and easings, e.g., they were only applicable after certain hours, grocery shopping was permitted, and essential workers could go to their jobs. This resulted in a discrepancy between the scenarios that would be valuable for policy makers and what was implemented by the modelers. This discrepancy could be attributed to a lack of collaboration during model development. Modellers have limited domain expertise and, thus, rely on policy actors and stakeholders to actively engage in participatory modelling, where they contribute with their expertise to define valuable scenarios and models (37).

These discrepancies are further complicated when there is a reliance on emerging digital health technology as a policy response to a health crisis. Here it is crucial not only to bridge the gap between the policy makers and the modellers, but to actively include domain experts in the everyday perspective of digital health technology for a range of technologies and groups within society. In so doing, the knowledge of the everyday experiences of past and current digital health technologies can support the scenario definition. By foregrounding the everyday in developing digital health interventions, the scenario development is enriched by the incorporation of the fluidity of the use, misuse, rejection or adaption of digital health technologies in the everyday. Centralising the everyday, and collaborating with a broad range of domain experts not only grounds scenarios in “real life,” but also serves to challenge the narrowing of scenarios of digital health in society by elite actors [see (38, 39)].

### 4.2 More appropriate data for model development

To be able to draw sound conclusions about the target system based on simulation results, it is necessary that the model correctly and realistically reflects the relevant parts of the target system. In simulations, verification and validation are applied to ensure the appropriateness and correctness of the model for a certain purpose (40). While verification ensures the correctness of the model’s implementation, validation assesses the applicability of the model and ensures it is an accurate representation of the target system. Both for the development but also for the validation of a simulation model, suitable data on the system and phenomenon that shall be studied is required, a lack of data might result in unrealistic models and results (41).

In ABSS, this need for appropriate data mainly concerns population data from the original population that is required for the generation of a synthetic population. This includes data on socio-demographic attributes of the population (e.g., age, gender, and household composition) but also data on the needs, desires, and goals of these individuals, which is required for appropriate modelling human decision making under different circumstances. Two common issues that modellers face include that data on specific attributes might be difficult to acquire and, if existing and accessible, this data often needs to be expanded and harmonized (42).

This demand of ABBS modellers can be seen as sitting between the social determinants of health and the everyday in digital health interventions in health crises. As Lupton (43) notes, numerous factors, such as income, education, location, age, disability, etc. impact the social structuring of digital health use. While these social determinants of health are vital to consider when developing and implementing public health crisis policy, everyday perspective of digital health can provide a wealth of data upon which to build models. Everyday digital health is largely a qualitative field, but there are examples of surveys and mixed methods approaches upon which to draw. This is facilitated by the self-collection and monitoring of data by “digitally engaged patients” [(44), p.256]. In addition, Stanley (45) describes data on the everyday as almost “naturally occurring” if we are to broaden our gaze to include already existing data sources. Given the time and resource restraints present during health crises, the ability to draw on already existing data is a significant advantage. One key aspect of this, which is related to the above opportunity, would be engagement with domain experts to help navigate this breadth of data, thus, mitigating the challenges which Chapuis and Taillandier (42) describe.

## 5 Summary

The rapid adoption of digital health technologies during and after the COVID-19 pandemic posed new challenges for policy makers. This paper argues that by accounting for the everyday digital health in modelling, ABSS can better support policy makers, and be a more powerful tool in future health crisis management. This claim in grounded in how the everyday digital health perspective can address two challenges of using ABSS identified during the COVID-19 pandemic, defining valuable scenarios and lack of appropriate data.

## Data availability statement

The original contributions presented in the study are included in the article/supplementary material, further inquiries can be directed to the corresponding author.

## Author contributions

JT: Writing – original draft. FL: Writing – original draft.

## Footnotes

^1^We use digital health as a broad and inclusive category where ICT and digital technology are involved in a range of health(care) arenas.

^2^Intelligent agents can be defined as autonomous entities that are characterised by individual attributes and that autonomously make decisions and perform actions based on their environment, individual situations, and attributes (9). In the context of ABSS, the goal of the agents is to imitate realistic human behaviour in order to create an artificial population that can be used for investigating complex phenomena within social systems (10).

^3^The everyday perspective has its foundations in sociology but has had far reaching applications across a range of disciplines. By focusing on the everyday, scholars "not only give importance to the ordinary, and take the ordinary seriously as a category of analysis, but they also evidence how everyday life, social relations, experiences and practices are always more than simply or straightforwardly mundane, ordinary and routine" [(32): 811].

## References

[1] Chen J Li K Zhang Z Li K Yu PS. A survey on applications of artificial intelligence in fighting against COVID-19. ACM Comput. Surv. (2021) 54:1–32. doi: 10.1145/3465398

[2] Rasheed J Jamil A Hameed AA Aftab U Aftab J Shah SA. A survey on artificial intelligence approaches in supporting frontline workers and decision makers for the COVID-19 pandemic. Chaos, Solitons Fractals. (2020) 141:110337. doi: 10.1016/j.chaos.2020.110337, PMID: 33071481 PMC7547637

[3] Patterson GE McIntyre KM Clough HE Rushton J. Societal impacts of pandemics: comparing COVID-19 with history to focus our response. Front Public Health. (2021) 9:630449. doi: 10.3389/fpubh.2021.630449, PMID: 33912529 PMC8072022

[4] Dron L Kalatharan V Gupta A Haggstrom J Zariffa N Morris AD. Data capture and sharing in the COVID-19 pandemic: a cause for concern. Lancet Digit Health. (2022) 4:e748–56. doi: 10.1016/S2589-7500(22)00147-9, PMID: 36150783 PMC9489064

[5] Iyamu I Gómez-Ramírez O Xu AX Chang HJ Watt S Mckee G. Challenges in the development of digital public health interventions and mapped solutions: findings from a scoping review. Digit. Health. (2022) 8:205520762211022. doi: 10.1177/20552076221102255 PMC9152201 35656283

[6] Scott BK Miller GT Fonda SJ Yeaw RE Gaudaen JC Pavliscsak HH. Advanced digital health technologies for COVID-19 and future emergencies. Telemed e-Health. (2020) 26:1226–33. doi: 10.1089/tmj.2020.0140 32456560

[7] Gilbert N Ahrweiler P Barbrook-Johnson P Narasimhan KP Wilkinson H. Computational modelling of public policy: reflections on practice. J Artif Soc Soc Simul. (2018) 21:14. doi: 10.18564/jasss.3669

[8] Hoad K Watts C. Are we there yet? Simulation modellers on what needs to be done to involve agent-based simulation in practical decision making. J. Simul. (2012) 6:67–70. doi: 10.1057/jos.2011.19

[9] Wooldridge M Jennings NR. Intelligent agents: theory and practice. Knowl Eng Rev. (1995) 10:115–52. doi: 10.1017/S0269888900008122

[10] Gilbert N. Agent-based social simulation: dealing with complexity. Complex Syst. Net. Excell. (2004) 9:1–14.

[11] Davidsson P. Agent based social simulation: a computer science view. J Artif Soc Soc Simul. (2002) 5.

[12] Lorig F Vanhée L Dignum F. Agent-based social simulation for policy making. In: M Chetouani V Dignum P Lukowicz C Sierra, editors. Human-centered artificial intelligence. ACAI 2021. Lecture notes in computer science. Cham: Springer (2023) 13500:391–414.

[13] Lorig F Johansson E Davidsson P. Agent-based social simulation of the COVID-19 pandemic: a systematic review. J Artif Soc Soc Simul. (2021) 24. doi: 10.18564/jasss.4601

[14] Banks J. Principles of simulation. In: J Banks, editor. Handbook of Simulation. New York: John Wiley & Sons (1998). 3–30.

[15] Winsberg E. Simulated experiments: methodology for a virtual world. Philos Sci. (2003) 70:105–25. doi: 10.1086/367872

[16] Conte R Paolucci M. On agent-based modeling and computational social science. Front Psychol. (2014) 5:668. doi: 10.3389/fpsyg.2014.00668 25071642 PMC4094840

[17] Kollmann M Sourjik V. In silico biology: from simulation to understanding. Curr Biol. (2007) 17:R132–4. doi: 10.1016/j.cub.2006.12.034 17307047

[18] Katsaliaki K Mustafee N. Applications of simulation within the healthcare context. J Oper Res Soc. (2011) 62:1431–51. doi: 10.1057/jors.2010.20, PMID: 32226177 PMC7099916

[19] Mielczarek B Uziałko-Mydlikowska J. Application of computer simulation modeling in the health care sector: a survey. SIMULATION. (2012) 88:197–216. doi: 10.1177/0037549710387802

[20] Tang J Vinayavekhin S Weeramongkolkul M Suksanon C Pattarapremcharoen K Thiwathittayanuphap S. Agent-based simulation and modeling of COVID-19 pandemic: a bibliometric analysis. J Disaster Res. (2022) 17:93–102. doi: 10.20965/jdr.2022.p0093

[21] Gilbert N Troitzsch K. Simulation for the social scientist. McGraw-Hill Education (2015).

[22] Dignum F Dignum V Davidsson P Ghorbani A van der Hurk M Jensen M. Analysing the combined health, social and economic impacts of the coronavirus pandemic using agent-based social simulation. Mind Mach. (2020) 30:177–94. doi: 10.1007/s11023-020-09527-6, PMID: 32836870 PMC7294191

[23] Fikar C Hirsch P Nolz PC. Agent-based simulation optimization for dynamic disaster relief distribution. CEJOR. (2018) 26:423–42. doi: 10.1007/s10100-017-0518-3

[24] Comis M Cleophas C Büsing C. Patients, primary care, and policy: agent-based simulation modeling for health care decision support. Health Care Manag Sci. (2021) 24:799–826. doi: 10.1007/s10729-021-09556-2, PMID: 34036444 PMC8147912

[25] Delmotte S Barbier JM Mouret JC Le Page C Wery J Chauvelon P. Participatory integrated assessment of scenarios for organic farming at different scales in Camargue, France. Agric Syst. (2016) 143:147–58. doi: 10.1016/j.agsy.2015.12.009

[26] Holmgren J Davidsson P Persson JA Ramstedt L. TAPAS: a multi-agent-based model for simulation of transport chains. Simul Model Pract Theory. (2012) 23:1–18. doi: 10.1016/j.simpat.2011.12.011

[27] Dignum F. (ed.). Social simulation for a crisis: Results and lessons from simulating the COVID-19 crisis computational social sciences Springer International Publishing (2021).

[28] Dignum F. Foundations of social simulations for crisis situations. In: Social simulation for a crisis: Results and lessons from simulating the COVID-19 crisis. Cham: Springer International Publishing (2021). 15–37.

[29] Jensen M Vanhée L Kammler C. Social simulations for crises: from theories to implementation. In: Social simulation for a crisis: Results and lessons from simulating the COVID-19 crisis. Cham: Springer International Publishing (2021). 39–84.

[30] Jensen M Lorig F Vanhée L Dignum F. Deployment and effects of an app for tracking and tracing contacts during the COVID-19 crisis. In: Dignum F, editor. Social simulation for a crisis: Results and lessons from simulating the COVID-19 crisis. Cham: Springer International Publishing (2021). 167–88.

[31] Davalbhakta S Advani S Kumar S Agarwal V Bhoyar S Fedirko E. A systematic review of smartphone applications available for corona virus disease 2019 (COVID19) and the assessment of their quality using the mobile application rating scale (MARS). J Med Syst. (2020) 44:1–15. doi: 10.1007/s10916-020-01633-3 PMC7417101 32779002

[32] Neal S Murji K. Sociologies of everyday life: editors’ introduction to the special issue. Sociology. (2015) 49:811–9. doi: 10.1177/0038038515602160

[33] Pink S Ruckenstein M Berg M Lupton D. Everyday automation setting a research agenda In: Pink S Berg M Lupton D Ruckenstein M, editors. Everyday automation: Experiencing and anticipating emerging technologies. New York: Routledge (2022). 1–19.

[34] Strange M Tucker J. AI and the everyday political economy of Global Health In: Lindgreen S, editor. The handbook of critical AI studies. Cheltenham: Edward Elgar (2023). 367–77.

[35] Wang Q Su M Zhang M Li R. Integrating digital technologies and public health to fight Covid-19 pandemic: key technologies, applications, challenges and outlook of digital healthcare. Int J Environ Res Public Health. (2021) 18:6053. doi: 10.3390/ijerph18116053, PMID: 34199831 PMC8200070

[36] Belfrage M. Lorig F. Davidsson P. (2022). Making sense of collaborative challenges in agent-based modelling for policy-making.

[37] Ahrweiler P Frank D Gilbert N. Co-designing social simulation models for policy advise: lessons learned from the INFSO-SKIN study. In: A D’Ambrogio F Barros X Hu, editors. 2019 spring simulation conference (SpringSim): IEEE (2019). 1–12.

[38] Hoff JL. Unavoidable futures? How governments articulate sociotechnical imaginaries of AI and healthcare services. Futures. (2023) 148:103131. doi: 10.1016/j.futures.2023.103131

[39] Tucker J. The future vision (s) of AI health in the Nordics: comparing the national AI strategies. Futures. (2023) 149:103154. doi: 10.1016/j.futures.2023.103154

[40] Balci. (2003). Verification, validation, and certification of modeling and simulation applications. In Proceedings of the 2003 winter simulation conference, 2003. (Vol. 1, pp. 150–158). IEEE.

[41] Kleijnen J. P. (1999). Validation of models: statistical techniques and data availability. In Proceedings of the 31st conference on winter simulation: Simulation---a bridge to the future-volume 1 (pp. 647–654).

[42] Chapuis K. Taillandier P. (2019). A brief review of synthetic population generation practices in agent-based social simulation. In submitted to SSC2019, Social Simulation Conference.

[43] Lupton D. Digital health: Critical and cross-disciplinary perspectives Routledge (2017).

[44] Lupton D. The digitally engaged patient: self-monitoring and self-care in the digital health era. Soc Theory Health. (2013) 11:256–70. doi: 10.1057/sth.2013.10

[45] Stanley L ed. Documents of life revisited: Narrative and biographical methodology for a 21st century humanism. Farnham: Ashgate (2013).

